# The arms race between bacteria CBASS and bacteriophages

**DOI:** 10.3389/fimmu.2023.1224341

**Published:** 2023-07-28

**Authors:** Lan Wang, Leiliang Zhang

**Affiliations:** ^1^Department of Clinical Laboratory Medicine, The First Affiliated Hospital of Shandong First Medical University and Shandong Provincial Qianfoshan Hospital, Jinan, Shandong, China; ^2^Medical Science and Technology Innovation Center, Shandong First Medical University and Shandong Academy of Medical Sciences, Jinan, Shandong, China; ^3^Department of Pathogen Biology, School of Clinical and Basic Medical Sciences, Shandong First Medical University and Shandong Academy of Medical Sciences, Jinan, Shandong, China

**Keywords:** CBASS, cell suicide, CD-NTase, immune evasion, phage

## Abstract

The Bacterial Cyclic oligonucleotide-Based Anti-phage Signaling System (CBASS) is an innate immune system that induces cell suicide to defend against phage infections. This system relies on cGAS/DncV-like nucleotidyltransferases (CD-NTase) to synthesize cyclic oligonucleotides (cOs) and CD-NTase-associated proteins (Caps) to execute cell death through DNA cleavage, membrane damage, and NAD depletion, thereby inhibiting phage replication. Ancillary proteins expressed in CBASS, in combination with CD-NTase, ensure the normal synthesis of cOs and prepare CD-NTase for full activation by binding to phage genomes, proteins, or other unknown products. To counteract cell death induced by CBASS, phage genes encode immune evasion proteins that curb Cap recognition of cOs, allowing for phage replication, assembly, and propagation in bacterial cells. This review provides a comprehensive understanding of CBASS immunity, comparing it with different bacterial immune systems and highlighting the interplay between CBASS and phage. Additionally, it explores similar immune escape methods based on shared proteins and action mechanisms between prokaryotic and eukaryotic viruses.

## 1 Introduction

**Figure 1:**
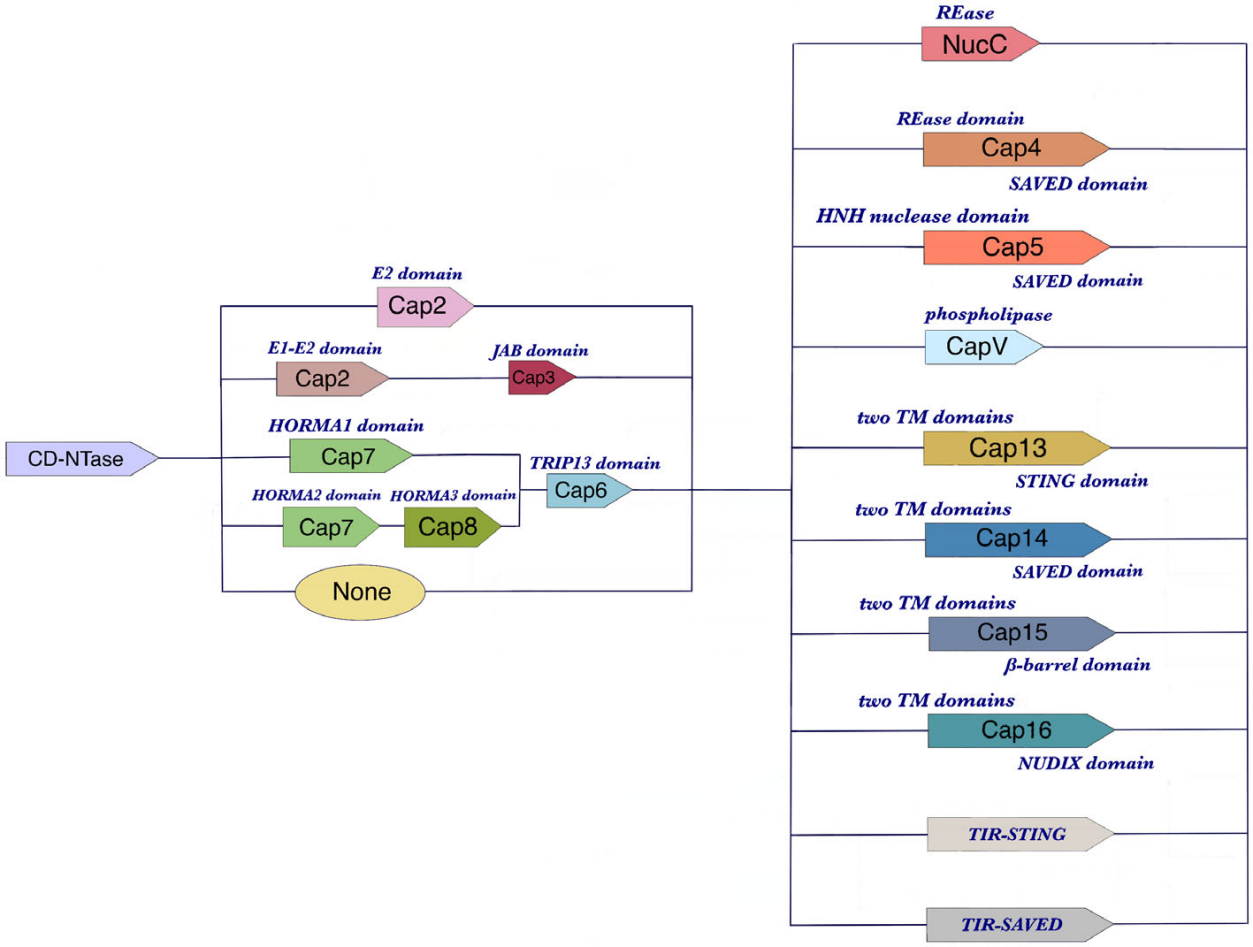
The components of CBASS operons. CBASS operons are genetic structures found in bacterial cells that are responsible for defending against phage infections. They typically consist of three types of genes: CD-NTase genes, ancillary genes, and effector genes. CD-NTase genes encode the CD-NTase enzyme, which is activated by phage genes or expression products to generate cyclic oligonucleotides (cOs). The ancillary genes produce proteins that help regulate the CD-NTase enzyme or activate the effector genes. Finally, the effector genes are responsible for inducing cell suicide and preventing phage replication. The specific arrangement and number of these genes can vary between different CBASS operons, but they all share this basic structure.

Cyclic guanosine monophosphate (GMP)-adenosine monophosphate (AMP) (cGAMP) synthase (cGAS), activated by viral or bacterial DNA in the cytoplasm of mammalian cells, can synthesize cGAMP as a second messenger to initiate Stimulator of Interferon Genes (STING) and ultimately induce the production of interferons (IFNs) to defend against virus invasion (1). Cyclic oligonucleotide-Based Anti-phage Signaling System (CBASS) has been identified as a common bacterial immune system arousing cell suicide through cyclic oligonucleotides (cOs) generated by cGAS/DncV-like nucleotidyltransferases (CD-NTases, similar to cGAS in eukaryotic cells) to activate CD-NTase-associated proteins (Caps) like CapV before completion of phage replication (2–4). Over 5,000 CBASS operons containing two or four gene systems commit diverse ancillary genes regulating CD-NTases activities, and express various effector cell-killing domains to perform DNA degradation, membrane destruction, or cellular metabolites depletion, leading to bacterial abortive infection (**Figure 1**) (2, 4).

Recent studies have found two anti-CBASS (Acb) proteins, Acb1 and Acb2, encoded by phage both directly target CD-NTase products cGAMP and then inhibit Cap effector activation to preserve cell survival and contribute to phage reproduction (2, 5). Moreover, NTases and MazG-like nucleotide pyrophosphatase, named Atd1, encoded by phage genome can block the Toll/interleukin-1 receptor (TIR)-STING pathway to ensure phage propagation (6). This phenomenon of virus antagonizing innate immune system is more diverse in virus-invading eukaryotic cells. For example, poxvirus immune nucleases (poxins), degrading 2’,3’-cGAMP, restrict the cGAS-STING pathway and inhibit the production of IFN (7). Prokaryotic and eukaryotic virus evolution in defending host immune systems present some similarities.

In this review, we will discuss the role of ancillary gene products in regulating CD-NTase activity, which is responsible for the synthesis of various cyclic oligonucleotides. Additionally, we will describe the structures and functions of the downstream Cap effectors that are modulated by multiple CBASS operons and compare their effector functions with CRISPR-Cas and Thoeris. Significantly, we will conclude on the recent studies about the structures and functional styles of proteins encoded in the phage genome that antagonize the CBASS system. We will also briefly illustrate the similarities of virus escaping immune attack between prokaryotic and eukaryotic cells.

## 2 The formidable phage defense strategies of CBASS

### 2.1 Cellular suicide

**Figure 2:**
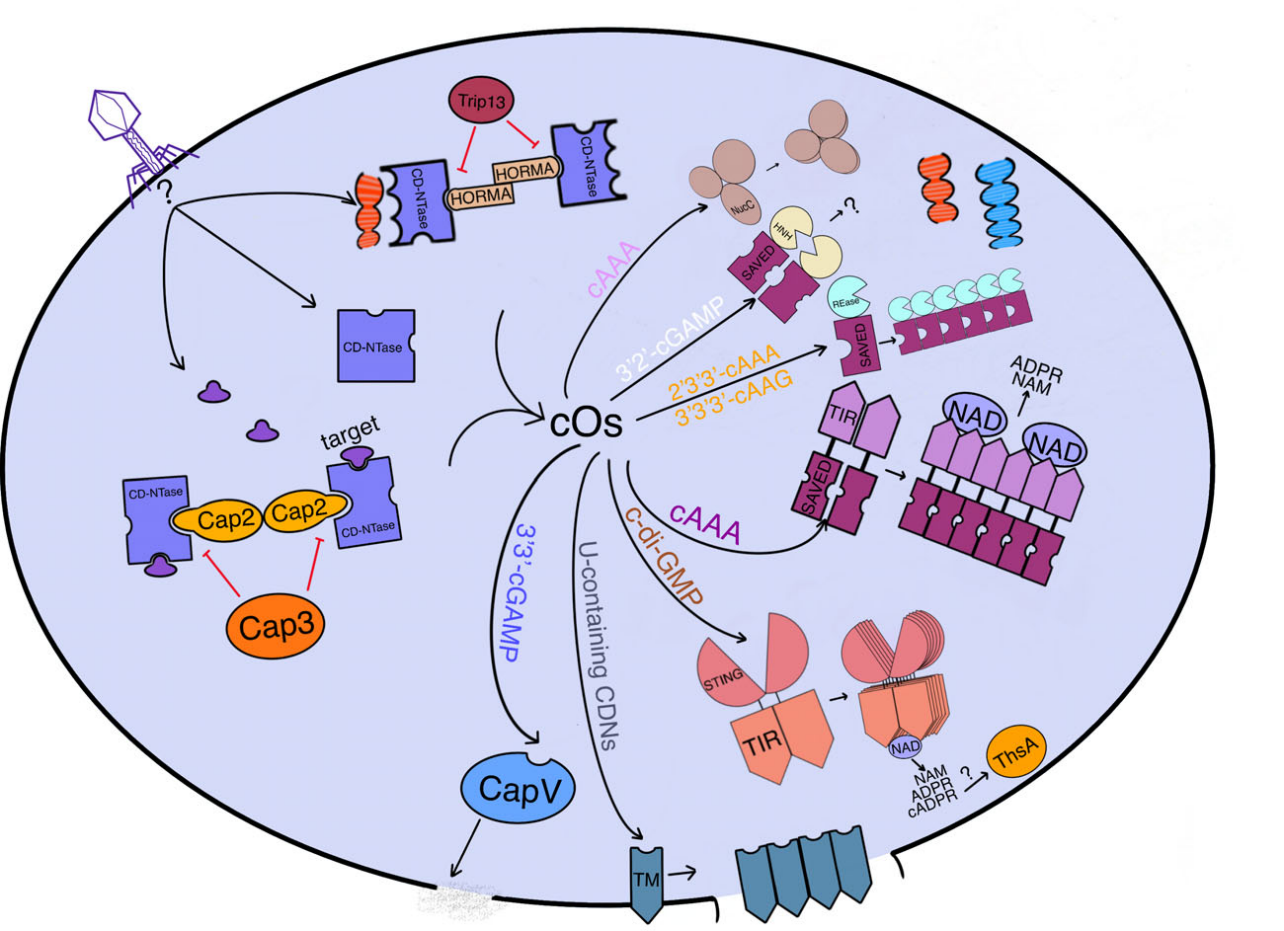
An overview of how CBASS immunity functions to prevent phage replication in bacterial cells. CBASS is a bacterial immune system that prevents phage replication by inducing cell suicide through various mechanisms. When a phage infects a bacterial cell, phage genes or expression products can activate the CD-NTase with or without accessory proteins (Cap2 or HORMA bind to CD-NTase while Cap3 or Trip13 disassemble the combination of CD-NTase and target proteins or accessory proteins), which generates cyclic oligonucleotides (cOs). Cap2 functions as an ubiquitinase to catalyze CD-NTase as the substrate to bind to the target for CD-NTase full activation. These cOs are recognized by the effector sensing domain, which activates effectors to implement cell suicide through DNA cleavage (endonucleases: NucC for cAAA, Cap4 for 2’3’3’-cAAA or 3’3’3’-cAAG, and Cap5 for 3’2’-cGAMP), membrane destruction (phospholipase CapV for 3’3’-cGAMP and TM-domain-containing Cap effectors for U-containing CDNs), or NAD degradation (NAD hydrolases: TIR-SAVED for cA_3_ and TIR-STING for c-di-GMP). Most effectors require oligomerization to fulfill their function. Meanwhile, ThsA from Thoeris may be activated by v-cADPR produced by TIR-STING. Overall, CBASS is an effective immune system that promotes the survival of bacterial hosts by preventing phage replication. Phage DNA is red while bacterial host DNA is blue.

The CBASS system has formidable phage defense strategies, one of which is cellular suicide through abortive infection (**Figure 2**). The CD-NTase synthesizes diverse and abundant cyclic di- and trinucleotides that are specific to the infected cells (3). These cyclic oligonucleotides function as second messengers that are sensed by Second Messenger Oligonucleotide or Dinucleotide Synthetase (SMODS)-associated and fused to various effector domains (SAVED), STING domains, and other cyclic oligonucleotide-sensing platforms of Caps. This activation of the Cap effector domain leads to the execution of cell suicide mainly through three individually classified effects, DNA degradation, membrane destruction, and cellular metabolites depletion (2, 8). Furthermore, the SAVED domain gene is widespread in Cap genes and co-exists with cell-killing domains, such as the HNH endonuclease domain, REase domain, TIR domain, calcineurin-like phosphoesterase domain, and two transmembrane helices (2TM) as the C-terminal cyclic oligonucleotide sensor of Cap effectors. The SAVED domain is capable of recognizing distinct cyclic oligonucleotide signals due to variations in its structure across different Caps (8, 9). Most CBASS operon configurations are composed of one cyclic oligonucleotide synthase and one Cap gene, which are named type I CBASS. Some CBASS operons have additional ancillary genes and are named type II/III CBASS (2). Heterogeneous Cap effectors accept host signals to promote cell death before the phage finishes a complete replication cycle, preventing the phage from spreading into the bystander bacteria.

### 2.2 DNA degradation

**Figure 3:**
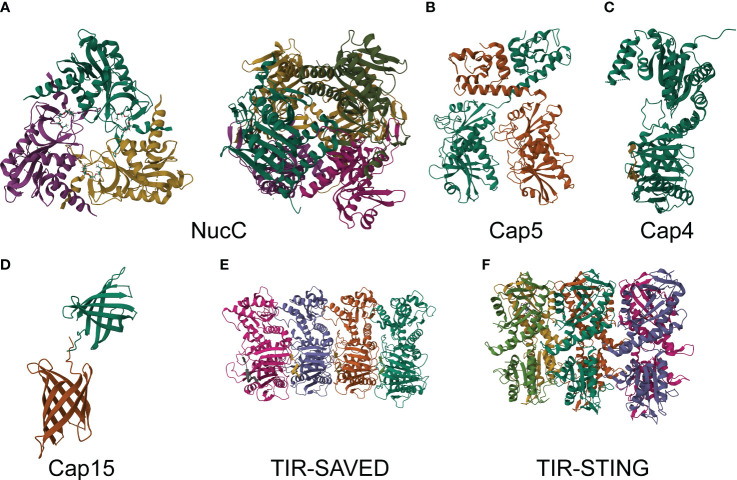
Structures of CBASS effectors. **(A)** Crystal structure of *E. coli* MS115-1 NucC trimer with cAAA in apo form at 1.75 Å resolution (PDB: 6P7O, left). Crystal structure of *E. coli* MS115-1 NucC hexamer with cAAA at 1.67 Å of resolution (PDB: 6P7P, right). **(B)** Crystal structure of *Asticcacaulis sp.* Cap5 at 2.39 Å of resolution (PDB: 7RWK). **(C)** Crystal structure of *Acinetobacter baumannii* Cap4 SAVED/CARF-domain containing receptor with 2’3’3’-cAAA at 2.10 Å of resolution (PDB: 6VM6). **(D)** Crystal structure of *Yersinia aleksiciae* Cap15 cyclic dinucleotide receptor of crystal form 2 at 2.60 Å of resolution (PDB: 7N35). **(E)** Crystal structure of *Microbacterium ketosireducens* TIR-SAVED effector bound to cA_3_ at 3.80 Å of resolution (PDB: 7QQK). **(F)** Crystal structure of *Sf*STING with c-di-GMP single fiber at 3.30 Å of resolution (PDB: 7UN8).

The nuclease effectors, including NucC, Cap4, and Cap5, are common endonuclease-related effectors that destroy host cellular DNA and phage genome to resist phage reproduction and implement abortive infection (**Figure 2**) (2). NucC is a monomer that is associated with the CD-NTase and contains a DNA endonuclease domain (**Figure 3A**) (8). In an inactive state, three NucC monomers form a homotrimer with an overall triangular architecture. The upstream signal cyclic AMP-AMP-AMP (cAAA) can activate the NucC trimer by binding to one trimer allosteric pocket with hydrogen bonds and π-stacking. Once activated, the NucC trimer conformationally changes, allowing two NucC trimers to interact with each other by hydrophobic clusters. The hexamer of the activated NucC sensitizes each NucC active site’s capacity to cleave one strand of DNA (10, 11). Another endonuclease with a high affinity for 3’2’-cGAMP, Cap5, consists of the SAVED domain and HNH nuclease domain, forming homodimers in an inactive state. In a homodimer, two SAVED domains recognizing 3’2’-cGAMP form an open and ajar configuration. Once 3’2’-cGAMP binds to one SAVED domain, it approaches the active site of the two HNH domains, which executes DNA degradation (**Figure 3B**) (12). Interestingly, both 3’2’-cGAMP and 2’3’-cGAMP have the 2’-5’ phosphate linkage which is generated by CD-NTase first. However, 2’-5’ phosphate linkage of 3’2’-cGAMP is composed of the 2’-OH of ATP and the α-phosphate of GTP, different from the 2'3'-cGAMP' s one composed of 2’OH of GTP and the α-phosphate of ATP. And the different 3’-5’ phosphate linkage consisting of ATP and GTP forms accordingly. 3’2’-cGAMP synthesized by bacterial CD-NTase functions as the activator of Cap5 for DNA degradation. Bacterial or viral DNA initiates cGAS to generate 2’3’-cGAMP, which can bind to STING and trigger the STING signaling pathway for resisting infection in mammalian cells (12, 13). Cap4 also possesses the SAVED domain for sensing cyclic oligonucleotides like Cap5 (**Figure 3C**). A recent research has shown that Cap4 proteins can be classified into two types based on the position of the cyclic oligonucleotide (cOs) binding site. Type I Cap4 proteins, such as AbCap4, position the cAAA molecule in the center of the SAVED domain (**Figure 3C**) (14). In contrast, type II Cap4 proteins, such as EcCap4, position the cAAG molecule near the N-terminal of the SAVED domain. This difference in cOs binding site location may contribute to the specificity of Cap4 proteins in different bacterial species (14). It is interesting to note that one SAVED domain formed by two CRISPR-associated Rossman fold (CARF) subunits of the Cap4 monomer recognizes the specific second messenger. Once activated, Cap4 can oligomerize into a high-order complex, which juxtaposes the active sites of the endonuclease domain, requiring ligand recognition. This oligomerization enforces its promiscuous nuclease activity for both host and phage genome (9, 14). In contrast to the endonucleases mentioned above, Cap18 has a 3’-5’ exonuclease primitive activity for degrading ssDNA or ssRNA, not requiring cyclic oligonucleotide binding. Coexisting with transcription factors such as CapW or CapH+CapP and Cap4 genes in CBASS operons, this Cap18 homodimer may regulate CBASS activation by eliminating ssDNA or ssRNA related to CBASS transcription factors (15). The bacterial CRISPR system also harbors a series of nuclease effector complexes for DNA or RNA damage. While NucC is present in both CBASS and some CRISPR systems, its function differs between the two. In CBASS, NucC induces cell suicide in response to DNA damage caused by the cytosine base editor. In CRISPR systems, NucC is involved in the degradation of foreign DNA by cleaving it into smaller fragments, which are then used by the CRISPR system to target and destroy the invading pathogen (16). NucC also has the ability to destroy bacteria chromosome for cell death instead of directly acting on phage genome (11, 17). Csx1 and Csm6 are ssRNases, and Can1 is a DNA endonuclease specific for cleaving ssRNA or dsDNA invasion, which does not cause abortive infection like CBASS (18–20). Card1 can not only directly destroy the phage genome due to its restriction endonuclease activity for just cleaving ssDNA or ssRNA but also provides an alternative to cell suicide (21). Different choices may depend on the degree of phage infection in bacteria with various immune systems, implying that the emergence of CBASS immunity may reflect phage infection has reached an irreversible point from an overall perspective (21). In summary, endonucleases leading to DNA damage possess unique activation conformations and special cyclic oligonucleotide second messengers, which may determine their specialty in different bacteria.

### 2.3 Membrane destruction

It is interesting to note that programmed cell death can be induced by membrane damage, which breaks the acid-base and osmotic balance (**Figure 2**). In *Vibrio cholerae*, for example, the bacteria harbor their own cGAMP synthase, DncV, which can generate 3’3’-cGAMP, specifically for activating CapV, a phospholipase that executes inner membrane degradation during phage infection (22). Genetic evidence has shown that 3’3’-cGAMP can directly bind to and activate CapV. Once activated, CapV degrades phosphatidylethanolamine (PE) and phosphatidylglycerol (PG) in the cell membrane, causing rapid cell death to inhibit phage reproduction. In addition to enzymatic activity, TM effectors can also disrupt the inner membrane and are encoded in more than 40% of CBASS operons. The most common TM effector is Cap15, which is composed of two N-terminal TM helices and a β-barrel C-terminal domain. The β-barrel domain functions as a uracil-containing cyclic dinucleotides sensor, forming a binding pocket (**Figure 3D**) (23). Once activated by cyclic dinucleotides (CDNs), Cap15 oligomerizes to form a high-order complex within the membrane, which is required for membrane damage. Interestingly, Cap15 without nucleotide second messenger recognition exists as an oligomeric complex rather than a monomer. Other important CBASS TM-containing effectors, such as Cap13, Cap14 (with a SAVED domain), and Cap16 (with a NUDIX domain), also produce cell toxicity through oligomerization to damage the inner membrane, accompanied by several other mechanisms (23). Thoeris ThsA effector has been identified as possessing an N-terminal transmembrane domain and a C-terminal NAD-binding domain, which is distinct from ThsA with the SIR2 domain executing NAD depletion (24). This transmembrane-domain ThsA may modulate cell death like membrane destruction caused by Cap15. Notably, Cap16 with a mutation of its NUDIX domain does not lose cell toxicity (23), implying that inner membrane destruction caused by TM effector oligomerization may be a simple but predominant strategy to induce rapid cell death for inhibiting phage replication and may be difficult for the phage to express certain proteins degrading or sequestering all kinds of uracil-containing cyclic dinucleotides.

### 2.4 NAD depletion

The TIR domain, which participates in NAD degradation, is widespread in different bacterial defense systems against phage invasion, such as CBASS and Thoeris (25). In CBASS, the TIR domain is fused to the SAVED or STING domain, which functions as the second messenger sensor. Upon phage infection, CD-NTase synthesizes 3’,3’,3’-cA_3_(same as 3’3’3’-cAAA and cAAA), which binds to each SAVED domain of two head-to-tail TIR-SAVED monomers to activate TIR-SAVED that ultimately forms a high-ordered filament (**Figures 2**, **3E**). Cryo-EM has identified this filament as a right-handed superhelical solenoid, with seventeen TIR-SAVED monomers in each turn of the filament. The TIR domain NADase active sites, enclosed by SAVED dimer formation, possess no NADase activity, implying the requirement of TIR-SAVED multimers for the highest NADase activity (26). Bacterial STING protein is homologous to the V-shaped human STING, but bacterial STING has no ability to recognize 2’-5’-linked cyclic dinucleotides, only specific for 3’-5’-linkage recognition. STING of TIR-STING has high affinity with c-di-GMP, binding to the central chamber formed by two STING interfaces as well as the TIR-STING homodimer (**Figure 3F**). Upon activation, TIR-STING undergoes conformational changes that result in the tight enclosure of c-di-GMP within the STING pocket. This, in turn, facilitates the formation of filaments through head-to-head contacts between STING proteins. The oligomerization of TIR-STING brings together the NADase active sites of TIR domains, allowing for the efficient degradation of nicotinamide adenine dinucleotide (NAD) into nicotinamide (NAM), ADP-ribose (ADPR), and a small amount of cyclic ADPR (cADPR). This unleashes the potent NADase activity of TIR-STING (27, 28). Both *Flavobacteriaceae sp.* STING (*Fs* STING) and *Capnocytophaga granulosa* STING (*Cg* STING) are known for their ability to recognize 3’3’-cGAMP instead of c-di-GMP, which is attributed to differences in their amino acid residues and β-strand lids. This similarity to eukaryotic STING suggests that there may be an evolutionary relationship between bacteria and animal cells (29). Unlike the CBASS operon, Thoeris only possesses two genes: ThsA as the NADase and ThsB as a TIR domain (30). Thoeris TIR domains possess nucleotide hydrolases and Toll-like receptors, but do not play a major role in degrading NAD (31). Phage invasion initiates TIR domain to generate an isomer of cADPR, as well as 3’cADPR (32), which is specific for NAD cleavage and can activate downstream ThsA SIR domains to execute NAD depletion (33). Interestingly, the production of 3’cADPR is required for TIR domain self-association (32). Moreover, eukaryotic SARM1 SAM domains can assemble into an octamer, triggering NAD cleavage by its TIR domain (34). Together, protein effectors oligomerization is significant for its function activation and rapid action for inhibiting phage or virus propagation. A recent study showed that NAD depletion can induce RelA expression and RelA synthesize (p)ppGpp, which initiates the mazFE toxin-antitoxin system to execute Abi, which is the same as starvation-inducing cell death. Atd1, which is a pyrophosphatase enzyme encoded by phages, has the ability to degrade (p)ppGpp, a signaling molecule that plays a role in regulating bacterial growth and stress responses. This degradation activity has been shown to hinder the abortive infection induced by the retron with TIR domain, which is a defense mechanism that some bacteria use to protect against phage infection (35). This phenomenon may suggest the possibility that multiple immune systems of bacteria are interconnected. Notably, previous research has identified some bacterial TIR domain proteins can cleave NAD into NAM, ADPR, and v-cADPR (36), implying bacteria may encode CBASS operons containing TIR domains producing v-cADPR along with ThsA homology, or oligomeric incomplete TIR domains of CBASS may produce 3’cADPR to activate ThsA. It is also possible that CBASS and Thoeris interact with each other to inhibit cell growth and terminate phage replication.

### 2.5 CBASS Starter: CD-NTase

CD-NTase is a cyclic oligonucleotide synthase with an unknown activation mechanism during phage infection. It is significant for CBASS immunity initiation due to its DNA polymerase β-like nucleotidyltransferase protein fold for the generation of cyclic di- or trinucleotides (2, 3). A thermo-denaturation assay has shown that certain combinations of nucleotides with donor pockets of CD-NTase (cGAS/DncV-like nucleotidyltransferase) can increase its thermo-stabilization, indicating that there is a specific reaction order for the nucleotides being catalyzed into cyclic oligonucleotides (37). The production of *Vibrio cholerae* enzyme dinucleotide cyclase in *Vibrio* (DncV) is not only significant for the efficiency of *Vibrio cholerae* intestinal colonization (21) but also imperative for CBASS defending against phage invasion through activating the effector phospholipase (CapV), executing inner membrane degradation for cell death (4). A four-gene CBASS operon considered as type II CBASS possesses DncV, CapV, and ancillary genes expressing ubiquitinase-like Cap2 containing an E1-E2 fusion domain and deubiquitylase-like Cap3 containing a JAB domain (4). Recent studies have identified that two Cap2 with adenylated E1 domains forming an all-in-one homodimer with two C-terminus of CD-NTase can brace CD-NTase activity by catalyzing CD-NTase as a substrate to bind a specific target protein and be indispensable for the CD-NTase full activation, existing in a four-gene CBASS during phage infection (38, 39). Interplay between CD-NTase and Cap2 resembles eukaryotic interaction between ubiquitin and E1 and E2 domain of ubiquitinase. Differently, a Cap2 cysteine residue forms a thioester with a conserved C-terminal glycine or alanine of CD-NTase, depending on ATP. Under Cap2 catalysis, the C-terminus of CD-NTase forms an isopeptide bond with one lysine of unknown phage proteins or other non-protein targets to completely activate CD-NTase itself. Interestingly, the C-terminus of cGAS still binds to Cap2 by thioester bonds with three cysteines and even isopeptide bonds with five lysines. cGAS conjugation enhances the production of second messengers, thus strengthening the antiviral response. As an isopeptidase, Cap3 possesses deconjugation activity for CD-NTase conjugation with activation factors, which can reduce the magnitude of the antiviral response. It is necessary for Cap2 and Cap3 to coexist integrally in their own CBASS for defending against some phages, implying that there are unknown benefits of deconjugation created by Cap3 (38, 39). The regulation of CD-NTase is also contained in type III CBASS, through HORMA (Hop1p, Rev7p, and Mad2) domain binding to CD-NTase to form a complex not upon phage infection and ATPase Pch2/TRIP13 for disassembling the active HORMA-CD-NTase complex. The activation of 2:2 HORMA-CD-NTase complex containing the HORMA1 domain depends on the presence of long double-stranded DNA, but the CBASS operons containing the HORMA2 and HORMA3 domain do not require dsDNA (10). Ancillary proteins encoded in the CBASS operon can upregulate or downregulate CD-NTase activity, thus controlling CBASS antiviral strength and probably preventing mistaken cell suicide induced by normal factors in the cytoplasm (**Figure 2**).

## 3 Phage self-salvation antagonizing CBASS immunity

### 3.1 Second messengers’ reduction

**Figure 4:**
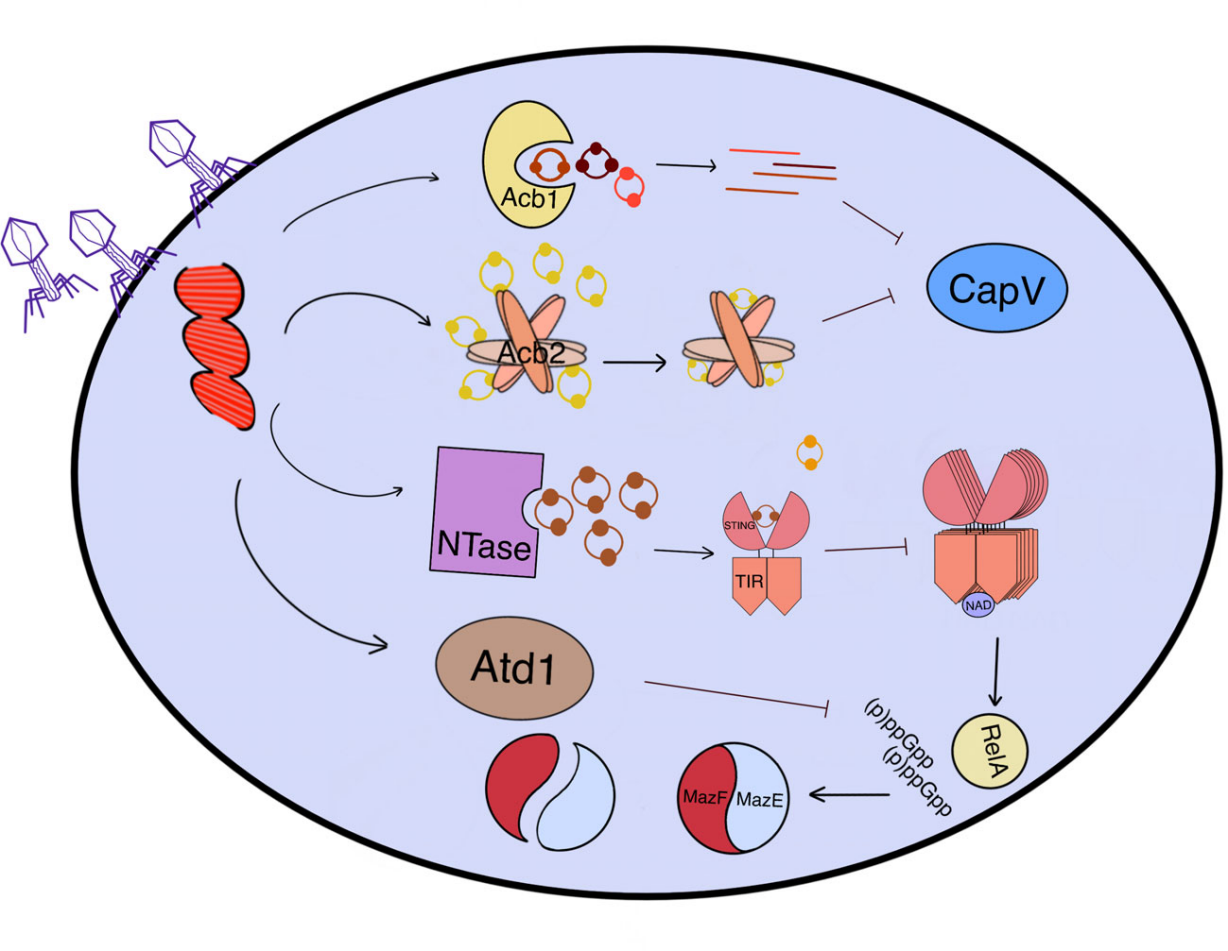
An overview of how phages counteract CBASS-induced cell suicide through various mechanisms. After the penetration of phages, the proteins expressed by phage genes (Red) in the cytoplasm can protect bacteria from cell death induced by CBASS effectors. Acb1 is a 3’-5’ bond hydrolase that transforms the ring oligonucleotide (3’3’-cGAMP, cUA, cAAA, and cAAG) into a linear one, while Acb2 sequesters cOs (2’3’-cGAMP, cAA, 3’3’-cGAMP, cUU, and cUA) like sponges, both of which can block effectors such as CapV. The phage NTase synthesizes the inhibitor 3’3’-cGAMP competing with 3’3’-cGG to bind to TIR-STING, thereby reducing the activity of TIR NADase. Moreover, MazG-like Atd1 can degrade (p)ppGpp and inhibit cell death caused by MazF toxicity by stabilizing the MazEF complex in an inactive state. All of these anti-CBASS (Acb) proteins work together to protect bacteria from cell suicide induced by phage infection.

Second messengers such as 3’,3’-cGAMP play a crucial role in activating Cap effectors that execute abortive infection in various ways to prevent phage replication. Recent studies have identified the anti-CBASS (Acb) protein encoded by the phage genome that can directly affect the cGAMP concentration in the cytoplasm (**Figure 4**) (5). The Acb1 gene was first identified as T4 phage gene 57B and can degrade CBASS signals 3’3’-cGAMP, cUA, cAAA, and cAAG, but not the non-canonical 2’-5’ phosphoesters, such as 3’2’-cGAMP. Moreover, the identification of 273 Acb1 phage proteins proves that Acb1 is a widespread form of phage escaping CBASS attack. The crystal structures of Acb1 are composed of six central β-strands, adopting a compact 2H phosphoesterase fold to form a U-shape ligand-binding pocket. The bond of cyclic dinucleotides or trinucleotides and Acb1 plays an essential role in stabilizing the rotated state of the adenosine base, having four conserved contacts and only needing one base-specific contact occurring between E141 and adenosine N6 position, illustrating the necessity of adenosine. This interaction can adjust the ligand to an optimal state liable to reposition the 2’OH for attack on the 3’-5’ bond. Located at an active-site HxT/HxT tetrad, the 3’ scissile phosphate of 3’3’-cGAMP adenosine can be used for acid-base catalysis and finally 3’3’-cGAMP is hydrolyzed into the linear product GpAp in a metal-independent reaction (5). The destruction of the cyclic state of normal CDNs terminates CBASS signaling in host defense, eventually protecting phage from abortive infection and promoting phage propagation.

In the experiment of dsDNA podophage PaMx41 invading *P. aeruginosa* strain (BWHPSA011; Pa011) with CBASS-dependent anti-phage immunity, the resistant PaMx41 possesses a no-stop mutation (X37Q) in orf24, which increases the length of gp24 (6). Through whole-genome sequencing, it has been found that the long gp24 , as the Acb2 protein, which is similar to Vs.4 from phage T4 and distinct from Acb1, is also present in other anti-CBASS phages such as PaMx33 (39). Acb2 is also encoded in many tailed phages and has a conserved function to inhibit CBASS immunity. Instead of combining with Cap effectors, Acb2 binds to 3’3’-cGAMP like a “sponge” to indirectly reduce CapV phospholipase activity, sequestering the CDNs. A previous study showed that Tad1 (Thoeris anti-defense 1) proteins encoded by phages are able to catch and sequester the signal molecule 1’–2’ glycocyclic ADPR (gcADPR) to preclude abortive infection (40). These two proteins differ from Acb1 which has the ability to hydrolyze CDNs and stabilize the molecule in an active state. The crystal structure of Acb2 in its apo form revealed that one Acb2 protomer comprises one short N-terminal helix and two long anti-parallel helices with a C-terminal kink, which contacts tightly with two nearby protomers to form a compact homo-hexamer. As one Acb2 homo-hexamer combines with three 3’3’-cGAMP, 3’3’-cGAMP binds to the pocket formed by the highly conserved N- and C-terminal helices/loops of two protomers interacting in a head-to-head manner, which is stabilized by hydrogen bonds, a salt bridge between protomers and phosphate groups of 3’3’-cGAMP, and hydrophobic interactions (6). Based on isothermal calorimetry (ITC), native gels, and high-performance liquid chromatography (HPLC) experiments, Acb2 can bind to 2’3’-cGAMP, cAA, 3’3’-cGAMP, cUU, and cUA with high affinity and can widely antagonize different kinds of CBASS types. Interestingly, phages with acb2 deletion have a missense point mutation in the major capsid gene, which assists phage to escape CBASS immunity. The wild-type major capsid gene is incapable of inducing CBASS-dependent cellular toxicity, while the mutated gene alone is insufficient to help the phage evade all CBASS immunity. The interaction mechanism between the major capsid protein and CBASS needs further study.

### 3.2 Blocking TIR-induced abortive infection by phage NTase and Atd1

TIR-STING is a protein that is activated by 3’3’-cGG, one of the downstream effectors of CBASS. It is a NADase that degrades NAD in the cytoplasm to induce cell suicide during phage infection. NTases, which are expressed by the phage genome in the cytoplasm, could generate a series of CDNs, including 3’3’-cGAMP, cAA, and cUA. The phage NTase synthesizes 3’3’-cGAMP in the bacterial cytoplasm, which competes with 3’3’-cGG for binding to TIR-STING. This competition reduces the activity of TIR NADase (**Figure 4**) (35). Adjacent to NTase genes, a phage Atd1 gene has been identified through plasmid-based functional screening as an additional antidefense gene. The expression product of Atd1 gene can degrade (p)ppGpp as a pyrophosphatase like MazG, which rescues MazE antitoxin expression and locks MazF toxicity (**Figure 4**). This stabilizes the MazEF complex in an inactive state, preventing cell death. In addition to Atd1, bioinformatics evidence has confirmed the presence of DNA methyltransferases and NAD synthase in phage genomes, which suggests that cell-killing domains may be deprived of their functions in DNA cleavage and NAD degradation during phage invasion (35). These proteins work together to inhibit cell suicide by hindering the activation or execution of effector functions.

## 4 Conclusion and prospect

It is fascinating to see the different strategies that viruses have evolved to suppress host immune responses, and the importance of innate immune responses in defending against these invasions. The CBASS system is a rapid and efficient cell-killing immunity that is activated by the generation of cOs by CD-NTase in response to phage cues. The full activation of CD-NTase in type II or III CBASS requires the presence of accessory proteins, and the decombining of these proteins is necessary for CD-NTase activity. The downstream Caps are activated by specific sensor parts that recognize cOs as second messengers, and these Caps implement cell death through various mechanisms to prevent phage replication, assembly, and propagation. It is interesting to note that some CBASS effectors process similar domains as Thoeris and CRISPR-Cas, and that phages have evolved mechanisms to prevent normal cOs or effectors from functioning. Understanding the interplay between CBASS and phage is important for developing a more accurate mechanism of CBASS immunity, exploring the evolution of innate immune responses in bacteria, and developing phage therapy. Continued research in this area will provide valuable insights into the complex interactions between viruses and their hosts.

CBASS immune system is widespread in bacteria, including three types of effectors to cause the cell death. However, differing from CBASS, CRISPR-Cas and Thoeris only possess one type of effector included in CBASS, the nuclease of CRISPS-Cas and NADase of Thoeris. Moreover, the existence of self-limiting *trans*-acting ring nucleases degrading cO such as Crn1 and Csx3 inhibit the activity of RNase or DNase for stabilizing cell survival (41), indicating there is a certain risk of recurrence of phage infection on this phenomenon. Individual effectors, second messengers, and accessory proteins can be ordered in different operons executing cell death in different ways, meaning that it is difficult for phage to completely inhibit all CBASS signal pathways in different bacteria. Once CBASS is activated, the infectious cell will suicide, which fundamentally suppresses the possibility of phage reproduction and propagation. Therefore, CBASS has great advantages over CRISPR and Thoeris because of its abundant diversity of operons and rapid cell-suicide mechanism.

Both bacterial CBASS and animal cGAS-STING are mechanisms of host innate immune system against virus invasion, in which CD-NTase and cyclic oligonucleotides play an essential role in initiating downstream effectors’ responses. Virus genes have evolved to antagonize host immunity. Similarly, poxins and Acb1 are both nuclease that cleave the 3’-5’ bond of cGAMP, thus suppressing host innate immunity (5, 7). As a viral nucleic acid sensor, eukaryotic cGAS could synthesize 2’, 3’-cGAMP, which activates STING on the ER and further induces IFN production to establish an antiviral state (42). However, the HSV-1 UL37 tegument protein acts as a deamidase to deamidate cGAS, aiming to abolish its ability to generate cGAMP, thereby inhibiting the cGAS-STING pathway (43). Additionally, the HSV-1 tegument protein UL41 is an RNase that degrades cGAS mRNA (44). Since CD-NTase and cGAS are homologs in structure and function (45), counteracting cGAS strategies by eukaryotic viruses may exist in a similar way during phage infection. The cleavage of STING by DTMUV NS2B3 protease activity can also be used as a reference strategy in CBASS, which contains the STING domain (46). A recent study has classified phage escape strategies, achieved through gene mutation, into three main categories: components of the phage core replication machinery, phage structural proteins, and host takeover mechanisms. This classification provides a foundation for new ideas on how phages evade CBASS immunity and which phage components sensitize CBASS starter CD-NTase (47).

Identifying the target of CD-NTase and the phage component recognized by CD-NTase is crucial to fully understand the mechanism of action of the CBASS system. It is possible that multiple phage proteins work together to activate CBASS (47). Additionally, understanding how the accessory proteins interact with each other to regulate CD-NTase activity is important to gain a better understanding of the overall function of the system. Further research is also needed to fully understand the interactions between CBASS and other immune systems and downstream effectors. Furthermore, additional research is needed to investigate whether the degradation of Acb2 or Tad1 proteins promotes phage release during the period when the phage is breaking down the host. Continued research in this area will provide valuable insights into the CBASS immune system and its potential applications in various fields, including medicine and biotechnology.

## Author contributions

LW wrote the first draft and generated the Figures. LZ conceived the study and revised the manuscript. All authors contributed to the article and approved the submitted version.
